# Computational methods in social neuroscience: recent advances, new tools and future directions

**DOI:** 10.1093/scan/nsab073

**Published:** 2021-06-24

**Authors:** Carolyn Parkinson

**Affiliations:** Department of Psychology, University of California, Los Angeles, CA 90095, USA

**Keywords:** computational social neuroscience, multivoxel pattern analysis, social network analysis, social decision-making, naturalistic neuroimaging

## Abstract

Recent years have seen a surge of exciting developments in the computational tools available to social neuroscientists. This paper highlights and synthesizes recent advances that have been enabled by the application of such tools, as well as methodological innovations likely to be of interest and utility to social neuroscientists, but that have been concentrated in other sub-fields. Papers in this special issue are emphasized—many of which contain instructive materials (e.g. tutorials and code) for researchers new to the highlighted methods. These include approaches for modeling social decisions, characterizing multivariate neural response patterns at varying spatial scales, using decoded neurofeedback to draw causal links between specific neural response patterns and psychological and behavioral phenomena, examining time-varying patterns of connectivity between brain regions, and characterizing the social networks in which social thought and behavior unfold in everyday life. By combining computational methods for characterizing participants’ rich social environments—at the levels of stimuli, paradigms and the webs of social relationships that surround people—with those for capturing the psychological processes that undergird social behavior and the wealth of information contained in neuroimaging datasets, social neuroscientists can gain new insights into how people create, understand and navigate their complex social worlds.

## Introduction

Creating, navigating and thriving within humans’ distinctively complex social environments are thought to entail some of the most significant computational challenges for the human brain and may have played a significant role in shaping its evolution (Dunbar, 2003). Scientific understanding of how the human brain meets such challenges is particularly likely to benefit from methodological tools that are capable of capturing the rich, multivariate and interdependent nature of the processes involved in social thought and behavior. Recent years have seen a surge of new developments in the methodological tools available to and used by social neuroscientists. Researchers studying how the human brain represents and navigates the social world increasingly integrate theory and methods from social psychology and neuroscience with computational approaches from network science, machine learning and other fields. There is also an abundance of methodological innovations from other areas of neuroscience that hold great promise for advancing our understanding of the social brain. This special issue, in tandem with other recent work in *Social Cognitive and Affective Neuroscience (SCAN)*, highlights and synthesizes advances in social neuroscience that have been enabled by the application of diverse computational methods, as well as methodological innovations likely to be of particular interest and utility to researchers studying how the brain supports social thought and behavior.

### Modeling social decisions and behaviors

To successfully navigate the social world, the human brain must perform a number of computations in parallel (e.g. tracking others’ behaviors, intentions, social identities and interactions, and using such information to shape one’s own behavior). In this issue, Molapour *et al.* (2021) outline a set of key computations with which the human brain must contend in any social interaction and emphasize the importance not only of examining each of these distinct computations individually, but also of understanding how the brain integrates information across computations and modalities to support social interactions. Examinations of how the brain supports social cognition and behavior using methods from computational neuroscience often involve creating a model of a particular social decision-making or learning process and relating parameters of that model to neural responses measured with functional magnetic resonance imaging (fMRI). For example, in this issue, Park *et al.* (2021) use this approach to shed light on the role of the right temporoparietal junction in updating impressions of close others and strangers. Much insight into the computations and mechanisms that guide human social thoughts, feelings and behavior has been achieved with such approaches. Also, in this issue Lockwood and Klein-Flügge (2021) provide a comprehensive and accessible primer on using one type of computational models (specifically, reinforcement learning models) to study social cognition. In this piece, Lockwood and Klein-Flügge (2021) describe theoretical and practical issues regarding the implementation of such models, promising directions for future research and ways in which applications of this approach can yield new hypotheses about how the human brain navigates everyday social situations. The authors also provide links to publicly available resources with tutorials and example code to assist readers interested in getting started with fitting reinforcement learning models to their data.

A growing body of computational social neuroscience research also uses approaches from ethology and behavioral ecology (Mobbs *et al.*, 2018). Such approaches contextualize social decisions in the kinds of scenarios that individuals regularly face in their natural social environment. In so doing, such research complements and extends findings that have been achieved using more constrained, and sometimes relatively decontextualized, paradigms. In this issue, Gabay and Apps (2021) provide an overview of one particular framework—marginal value theorem (Charnov, 1976)—that holds particular promise for examining questions about social cognition and behavior. Marginal value theorem characterizes decisions that individuals must make regarding when to abandon a current location and move onto a new setting when foraging for rewards. In addition to providing an overview of this framework, Gabay and Apps (2021) provide guidance for researchers seeking to implement such techniques in their own research and discuss how various aspects of social cognition and behavior can be conceptualized in this way (e.g. as foraging for social information), and thus, could be fruitfully studied through the lens of marginal value theorem. Continuing to integrate approaches from ethology and behavioral ecology into social neuroscience promises to enrich our understanding of the neural mechanisms underlying social thought and behavior by facilitating the precise quantification of decisions and behavior in increasingly complex situations that resemble those that individuals encounter in everyday life.

### Probing neural representations with multivariate approaches

#### Multivariate pattern analysis: fundamentals and methodological considerations.

Another area in which the integration of computational methods has expanded the questions that social neuroscientists can ask is multivoxel pattern analysis (MVPA) of fMRI data. Recent ‘Tools of the Trade’ articles in *SCAN* have provided comprehensive overviews of MVPA in general (Weaverdyck *et al.*, 2020) and of representational similarity analysis (RSA) in particular (Popal *et al.*, 2019). MVPA approaches can afford sensitivity to information carried in distributed neural response patterns, provide insight into how particular brain regions organize information (i.e. what rules govern which stimuli or mental states are treated as relatively similar or distinct from one another, in terms of evoked neural response patterns), and elucidate the significance of overlapping activations across different tasks, domains or contexts (Weaverdyck *et al.*, 2020). There are many important considerations for design, analysis and interpretation that should be taken into account when using MVPA, as discussed in depth in the aforementioned papers (Popal *et al.*, 2019; Weaverdyck *et al.*, 2020). One particular consideration pertains to the spatial scale of the representations targeted for study, as discussed briefly in Weaverdyck *et al.* (2020) and in great detail in this issue by Jolly and Chang (2021). Of note, Jolly and Chang (2021) discuss the assumptions and consequences associated with using MVPA approaches that are sensitive to effects carried at different spatial scales (e.g. searchlight analyses, region-of-interest analyses and whole-brain predictive modeling). They also provide concrete guidelines regarding how researchers can make an informed choice about which analytic technique to use in order to afford maximal sensitivity to the effects that they aim to capture.

#### Using MVPA to answer questions of interest to social neuroscientists.

This issue also contains examples of how MVPA methods can be applied to answer questions of particular interest to social neuroscientists. For example, Thornton and Tamir (2021) examine the multivoxel response patterns evoked when viewing naturalistic action sequences and suggest a framework (the ACT-FAST model) that the brain may use to represent others’ actions that are currently being observed and to predict future actions. Additionally, Londerée and Wagner (2021) apply RSA to demonstrate that the orbitofrontal cortex encodes multiple dimensions of value for a particular class of stimuli (here, the tastiness and health value of food) and demonstrate that this region contains finer-grained representations of relative tastiness at the upper end of that dimension. While Londerée and Wagner (2021) focus on non-social appetitive stimuli (food), there is great promise in applying such an approach when probing the representation of other people. For example, what dimensions organize our representations of social partners during interactions and how might this change across contexts? What determines which people are represented as particularly distinct from one another? Do we always simultaneously encode multiple dimensions of social value when encountering other people? How might the answers to these questions differ across brain regions?

More generally, Brooks *et al.* (2021) review how the application of MVPA to fMRI data has afforded insights into the neural basis of social perception. For example, by elucidating the ways in which different brain regions’ representations are organized, MVPA can shed light on how social information is transformed at different stages of processing and how particular brain regions may undergird particular social-perceptual processes.

#### Establishing causal links between brain and behavior.

An often-noted limitation of functional neuroimaging methods like fMRI is that such methods only allow for establishing correlational relationships between neural and psychological and/or behavioral phenomena. However, an exciting new approach leverages MVPA methods to allow researchers to establish causal links between particular brain states and particular cognitive, affective and behavioral outcomes: fMRI-based decoded neurofeedback. In this issue, Taschereau-Dumouchel *et al.* (2021) provide an accessible overview of this approach and guidelines for researchers seeking to employ the technique in their own research.

Briefly, in decoded neurofeedback studies, researchers target occurrences of a particular multivoxel response pattern by providing participants with real-time feedback about the activation likelihood of that response pattern, which is also associated with a reward (and, in some cases, the presentation of particular stimuli, to create new associations between the targeted pattern and such stimuli). Participants are not provided with any particular training strategy to evoke the desired patterns and, thus, can be kept unaware of the phenomena with which the elicited patterns are typically associated (e.g. participants could be taught to evoke a neural response pattern associated with seeing a spider without ever knowing that the response pattern that they are evoking typically accompanies seeing a spider). Participants then show specific and robust effects on physiological and behavioral outcomes related to the targeted neural representations. For example, in the aforementioned example, the participant’s fear of spiders would be reduced after repeatedly eliciting neural representations similar to those evoked when viewing spiders, without their awareness of the meaning of the neural patterns that they had evoked (Koizumi *et al.*, 2017; Taschereau-Dumouchel *et al.*, 2018). Across a range of recent studies, researchers have directly and unconsciously shaped a variety of psychological processes (e.g. metacognition, threat reactivity, learning, and emotion perception) by targeting specific neural response patterns through decoded neurofeedback (Taschereau-Dumouchel *et al.*, 2021).

The exciting implications of decoded neurofeedback are not limited to therapeutic interventions (e.g. fear reduction, Koizumi *et al.*, 2017; Taschereau-Dumouchel *et al.*, 2018). This approach also establishes that the induced response patterns caused the observed psychological outcomes, rather than merely establishing a correlational link. Researchers have begun to apply this approach to study social phenomena in a small handful of studies (e.g. Moll *et al.*, 2014; Shibata *et al.*, 2016; Ramot *et al.*, 2017) and holds great promise, particularly given that other methods for establishing causal links between neural and psychological phenomena in humans do so at a much coarser level of granularity. For example, whereas anatomical lesion studies or transcranial magnetic stimulation can causally link entire brain regions to particular psychological or behavioral phenomena, decoded neurofeedback approaches can reveal the causal effects of specific multivoxel response patterns (e.g. Taschereau-Dumouchel *et al.*, 2018) or specific functional connectivity patterns (e.g. Ramot *et al.*, 2017) on particular psychological and behavioral phenomena.

### Connectivity and the social brain

Recent advances in social neuroscience have also involved characterizing functional and structural connectivity between brain regions. These have included functional connectome-based ‘fingerprinting’ methods that characterize patterns of functional connectivity between brain regions that are diagnostic of individual identity (Finn *et al.*, 2015) and that are also predictive of various traits (e.g. fluid intelligence, attentional abilities and personality traits, Finn *et al.*, 2015; Rosenberg *et al.*, 2016; Hsu *et al.*, 2018), dimensions of psychopathology (Xia *et al.*, 2018), and people’s social relationships with one another (Hyon *et al.*, 2020b). Other methods for characterizing functional and structural brain that are particularly promising for future social neuroscience applications include examinations of time-varying functional connectivity (Calhoun *et al.*, 2014), which can capture how brain states evolve over time, as well as edge-centric functional network representations, which can capture how connections between brain regions interact with one another (Faskowitz *et al.*, 2020), and multimodal approaches that demonstrate, for example, how the functional profiles of brain regions relate to their underlying patterns of structural connectivity (Saygin *et al.*, 2016; Tovar and Chavez, 2021).

This issue contains two articles focused on a subset of the methods described above, with concrete guidance on how social neuroscience researchers new to such methods can apply them in their own research. First, a ‘Tools of the Trade’ article by Iraji *et al.* (2021) provides a gentle introduction to time-varying connectivity methods, including guidance on how to use an openly accessible toolbox for capturing properties of dynamic functional connectivity and on interpreting measures that are calculated in such analyses. Second, Tovar and Chavez (2021) link the functional organization of the medial prefrontal cortex (based on patterns of functional coactivation) to its structural organization (based on patterns of structural connectivity) and find similar parcellations of this region using both approaches. These results shed light on the internal organization of a brain region that is consistently implicated in social and affective phenomena—the medial prefrontal cortex (Lieberman *et al.*, 2019)—and provide evidence in support of the notion that the structural connectivity of brain regions constrains their functional profiles. The authors supplement their empirical article with an online tutorial to facilitate the adoption of such approaches by other researchers.

While functional connectivity is often characterized during rest (i.e. in the absence of external stimulation), a growing body of research highlights the value of characterizing patterns of functional connectivity during particular kinds of naturalistic stimulation (e.g. while viewing audiovisual movies). Using data acquired during movie-viewing, rather than rest, yields more reliable estimates of functional connectivity (Wang *et al.*, 2017) that are more predictive of trait-level variables than resting-state functional connectivity (Finn and Bandettini, 2021), particularly when participants are shown movies that are rich in social content. Thus, future work could likely productively extend this approach to gain insight into how individual differences in functional connectivity between brain regions relate to individual differences in how people interact with, think about and emotionally respond to one another.

### Examining the brain in its social context

#### Naturalistic stimuli.

In recent years, there has been considerable interest in examining the neural basis of social and affective processes in contexts that more closely resemble the rich, dynamic perceptual experiences that populate everyday life than traditional fMRI paradigms. The tasks that have traditionally been used in many functional neuroimaging studies have tended to prioritize experimental control, which has often entailed stripping stimuli of their surrounding context (e.g. by presenting participants with images or trials of particular tasks, preceded and followed by fixation crosses or blank screens, Sonkusare *et al.*, 2019). Such approaches have yielded, and continue to yield, many important insights about human brain function. In contrast, naturalistic neuroimaging studies often use exceptionally engaging, context-rich stimuli where narrative meaning unfolds over time (e.g. audiovisual movies and audio recordings of short stories, Sonkusare *et al.*, 2019). Because data from each time point are inextricably linked to its surrounding temporal and narrative context in such paradigms, and since many stimulus-level features often vary in tandem with one another within and across naturalistic stimuli, it is often difficult to ascertain what aspects of stimuli, paradigms or evoked mental processes drive observed effects or to isolate targeted perceptual, cognitive or affective constructs. However, while naturalistic paradigms sacrifice experimental control, they also yield significant benefits, such as increased ecological validity and the ability to study how stimuli are processed when embedded in the narrative and temporal contexts that typically surround them. Examining how brain activity evolves over time in response to complex, dynamic stimuli in which meaning unfolds over the course of minutes or hours, rather than milliseconds, may be particularly valuable for social neuroscientists, given that many of the brain regions that play important roles in social cognition (e.g. regions of the default mode network) have relatively long temporal receptive windows—i.e. are attuned to information that unfolds over relatively long periods of time (Hasson *et al.*, 2008). Thus, the stimuli and data analytic approaches often used in naturalistic neuroimaging studies are particularly well-suited to studying information processing within these regions and the aspects of social functioning that they support.

Researchers often analyze data from naturalistic fMRI studies by linking inter-subject correlations of time series of neural response magnitudes to similarities in subjective understanding, emotional responding, memory or other outcomes (e.g. Yeshurun *et al.*, 2017; Nguyen *et al.*, 2018); see Nastase *et al.* (2019) for a recent overview of methodological considerations for inter-subject correlation analyses. Recent work has also examined how multivoxel response patterns fluctuate over time (e.g. Baldassano *et al.*, 2017; Hyon *et al.*, 2020a; Chang *et al.*, 2021), as well as patterns of functional connectivity (e.g. Finn and Bandettini, 2021), during naturalistic stimulation.

Inter-subject similarities of neural response time series during naturalistic stimulation have been linked to individual difference variables related to social cognition, such as trait paranoia (Finn *et al.*, 2018) and the development of theory of mind (Richardson *et al.*, 2018), as well as to friendships and the proximity between people in their real-world social networks (Parkinson *et al.*, 2018; Hyon *et al.*, 2020a). Further underscoring the relevance of this approach to social neuroscientists, as described in the preceding section (‘Connectivity and the social brain’), patterns of functional connectivity evoked during naturalistic stimulation are most successful in predicting trait-level variables when the naturalistic stimuli used are rich with social content, such as people, faces and dialog (Finn and Bandettini, 2021).

#### Social interactions.

Another characteristic that distinguishes how social cognitive processes unfold in everyday life from how they have often been studied in neuroimaging research is their interactive nature. Even when participants in fMRI studies view social stimuli or perform social tasks, they often do so while in a room (e.g. a scanner suite) by themselves, in the absence of social interaction. Sometimes, participants interact with real or virtual social partners who are located outside of the scanner (Redcay *et al.*, 2010), allowing researchers to examine one half of a dyadic social interaction, which is sufficient to fruitfully examine many phenomena, such as the neural basis of making socially guided decisions during interactions (Carter *et al.*, 2012). Examining how brains dynamically interact during social interactions, however, demands different approaches to data acquisition and analysis.

From the perspective of data acquisition, some studies have adopted hyper-scanning approaches to fMRI, where two or more participants can communicate with each other while being scanned simultaneously (for a recent review, see Misaki *et al.*, 2021); others have examined inter-brain coupling in social contexts using electroencephalography (e.g. Dumas *et al.*, 2010; Dikker *et al.*, 2017; Goldstein *et al.*, 2018) and functional near-infrared spectroscopy (fNIRS) (for a recent review, see Burns and Lieberman, 2019). fNIRS is a particularly promising method for studying social interactions in contexts that bear a strong resemblance to everyday life, given that participants can move, express themselves and interact relatively freely, allowing for more natural behavior. Moreover, fNIRS equipment is often quite portable, affording the ability to collect data in a greater diversity of settings than most other neuroimaging modalities would allow (Burns *et al.*, 2019; Burns and Lieberman, 2019).

From the perspective of data analysis, while many studies have looked at inter-subject correlations of neural response time series during social interactions, mirroring common practices in naturalistic neuroimaging more generally, it is clear that such an approach does not capture all aspects of interpersonal coupling that take place during social interactions (Hasson and Frith, 2016). Advancing our understanding of how the human brain supports dynamic, reciprocal social interactions will require the further adoption and development of data analytic approaches that are capable of capturing other aspects of interpersonal complementarity during social interactions and relating such complementarity to properties of social interactions, to the construction of shared meaning, and to other individual-, dyad- and group-level outcomes; see Redcay and Schilbach (2019) for a thoughtful recent discussion of relevant issues and methods.

#### Social networks.

A growing body of research also integrates approaches from social neuroscience for characterizing information processing within individual brains with approaches for characterizing the social networks that people inhabit (e.g. Zerubavel *et al.*, 2015; Parkinson *et al.*, 2017; Schmälzle *et al.*, 2017; Peer *et al.*, 2021); for recent reviews, see Falk and Bassett (2017) and Weaverdyck and Parkinson (2018). Integrating approaches for collecting and analyzing real-world social network data into social neuroscience can provide opportunities to gain insight into how people understand, shape and are shaped by the structure of their social worlds. In this issue, Baek *et al.* (2021) provide an overview for social neuroscientists of key theoretical and methodological issues in social network analysis, with examples of how approaches from social neuroscience and social network analysis can be productively combined, tutorials to aid in the practical implementation of many key concepts, and a discussion of outstanding issues and questions in this area of research.

Approaches using naturalistic stimuli, interactive paradigms and characterizations of real-world social networks can greatly enrich our understanding of how the brain supports social processes in everyday life. By taking into account more aspects of the contexts in which social processing unfolds in the real world, such approaches promise to complement the continued use of more constrained experimental paradigms that afford greater internal validity and that have provided much of the foundational knowledge on which the research discussed in this section has been built.

## Conclusions

The set of computational tools employed in social neuroscience continues to grow and advance. Such tools provide new ways to richly characterize the psychological processes that underlie social decisions, neural responses at different levels of granularity, and the real-world social contexts in which social cognition and behavior unfold. Efforts to continue to advance our understanding of the neural basis of social thought and behavior will no doubt benefit from integrative approaches, both in terms of examining how different cues and computations are integrated by the brain to support social interactions (Molapour *et al.*, 2021) and in terms of integrating computational approaches that capture the information contained in multivariate neural data, in modeled approximations of social thought and behavior, and in our complex social surroundings. Such interdisciplinary endeavors are increasingly recognized as necessary for addressing fundamental questions about human behavior (for a thoughtful recent discussion of the challenges associated with the increasingly interdisciplinary nature of social science and potential solutions to such challenges, see Buyalskaya *et al.*, 2021). Continuing to adopt, develop and combine computational methods for characterizing the patterns of social ties that surround people, the psychological processes supporting social behavior and the wealth of information contained in neuroimaging data promises to advance our understanding of how people create, make sense of and navigate their social worlds.
